# Translating Suicide Safety Planning Components Into the Design of mHealth App Features: Systematic Review

**DOI:** 10.2196/52763

**Published:** 2024-03-28

**Authors:** Kim Gryglewicz, Victoria L Orr, Marissa J McNeil, Lindsay A Taliaferro, Serenea Hines, Taylor L Duffy, Pamela J Wisniewski

**Affiliations:** 1 School of Social Work University of Central Florida Orlando, FL United States; 2 Center for Behavioral Health Research & Training University of Central Florida Orlando, FL United States; 3 Department of Population Health Sciences University of Central Florida Orlando, FL United States; 4 Department of Computer Science Vanderbilt University Nashville, TN United States

**Keywords:** suicide prevention, suicide safety planning, mobile health, mHealth apps, eHealth, digital health, systematic review, Preferred Reporting Items for Systematic Reviews and Meta-Analyses, PRISMA

## Abstract

**Background:**

Suicide safety planning is an evidence-based approach used to help individuals identify strategies to keep themselves safe during a mental health crisis. This study systematically reviewed the literature focused on mobile health (mHealth) suicide safety planning apps.

**Objective:**

This study aims to evaluate the extent to which apps integrated components of the safety planning intervention (SPI), and if so, how these safety planning components were integrated into the design-based features of the apps.

**Methods:**

Following the PRISMA (Preferred Reporting Items for Systematic Reviews and Meta-Analyses) guidelines, we systematically analyzed 14 peer-reviewed studies specific to mHealth apps for suicide safety planning. We conducted an analysis of the literature to evaluate how the apps incorporated SPI components and examined similarities and differences among the apps by conducting a comparative analysis of app features. An independent review of SPI components and app features was conducted by downloading the available apps.

**Results:**

Most of the mHealth apps (5/7, 71%) integrated SPI components and provided customizable features that expanded upon traditional paper-based safety planning processes. App design features were categorized into 5 themes, including interactive features, individualized user experiences, interface design, guidance and training, and privacy and sharing. All apps included access to community supports and revisable safety plans. Fewer mHealth apps (3/7, 43%) included interactive features, such as associating coping strategies with specific stressors. Most studies (10/14, 71%) examined the usability, feasibility, and acceptability of the safety planning mHealth apps. Usability findings were generally positive, as users often found these apps easy to use and visually appealing. In terms of feasibility, users preferred using mHealth apps during times of crisis, but the continuous use of the apps outside of crisis situations received less support. Few studies (4/14, 29%) examined the effectiveness of mHealth apps for suicide-related outcomes. Positive shifts in attitudes and desire to live, improved coping strategies, enhanced emotional stability, and a decrease in suicidal thoughts or self-harm behaviors were examined in these studies.

**Conclusions:**

Our study highlights the need for researchers, clinicians, and app designers to continue to work together to align evidence-based research on mHealth suicide safety planning apps with lessons learned for how to best deliver these technologies to end users. Our review brings to light mHealth suicide safety planning strategies needing further development and testing, such as lethal means guidance, collaborative safety planning, and the opportunity to embed more interactive features that leverage the advanced capabilities of technology to improve client outcomes as well as foster sustained user engagement beyond a crisis. Although preliminary evidence shows that these apps may help to mitigate suicide risk, clinical trials with larger sample sizes and more robust research designs are needed to validate their efficacy before the widespread adoption and use.

## Introduction

### Background

Suicide is one of the leading causes of death in the United States, accounting for >45,000 deaths annually [1]. Over the last decade, suicide rates have doubled for youth aged 10 to 24 years [2] and have steadily increased for racial and ethnic minority youth [1,3,4]. Suicide ideation and attempt rates have also risen [5,6], especially among youth and minoritized populations [5,7-11]. Numerous studies have shown that untreated mental illness, limited or lack of available care, and low perceived need for mental health treatment are common, yet preventable, suicide risk antecedents [12-19]. Moreover, stigma, difficulties recognizing suicide warning signs, preferences for self-reliance and autonomy, fear of burdening others, and negative treatment experiences can negatively affect help-seeking intentions and engagement in mental health services [20-24].

Researchers have identified various suicide prevention strategies to reduce the public health problem of suicide [25,26]. Safety planning is an integral component of suicide care [27] and has been empirically validated for reducing suicidality [28,29]. The process of safety planning involves collaboration between a clinical and client, as well as with the at-risk individual and their support network. This means that the support network could also be part of the safety planning process [30]. Safety planning involves jointly identifying, problem-solving, and communicating strategies to keep an individual safe if a crisis arises. Core strategies focus on uncovering warning signs or triggers that precede an emotional event, identifying and reinforcing the use of healthful self-management strategies to cope with distress, encouraging the use of positive socialization strategies for distraction and support, creating a network of external support and professional contacts to solicit assistance and support, and reducing access to lethal means [31]. The individualized nature of creating a safety plan (ie, a written document detailing the plan to keep an individual safe during a crisis) allows the person at risk of suicide the ability to incorporate culturally relevant and meaningful strategies, thereby making these plans useful and relevant for diverse populations [30,32].

Suicide safety planning is a brief intervention that has been used in both acute and clinical settings [31,33,34] and as a self-help tool [35]. Overall, researchers have found this intervention to be feasible, acceptable, and useful to facilitate support and reduce suicide risk [32,33,35-37]. Researchers have found safety plans and related interventions, such as crisis response planning [38], to be effective in reducing the risk of hospitalization, increasing engagement in mental health treatment, and promoting the use of healthful coping strategies when used alongside other therapeutic approaches [33,34,36,39,40]. Although safety planning has shown initial success in reducing suicidal urges and offering a sense of hope to individuals in crisis [41], some clinicians and researchers have criticized this process [42,43]. For example, safety planning encourages clinicians to revisit and update safety plans with their clients over time [44], which can prove challenging if service use barriers prevent clients from reaccessing care or if clients misplace or throw away their paper-based safety plan.

Considering these challenges, mobile health (mHealth) technologies could offer a timely and effective solution to address some of the criticisms directed at traditional safety planning methods. mHealth, particularly the use of apps, represents a common tool used by consumers with access to mobile phones [45,46]. In addition, mHealth has garnered attention as a practical and convenient method for implementing mental health interventions [47], with increase in the quantity and functionality of applications and tools resulting in increased use [48]. In general, mHealth apps have been used to effectively help individuals identify and manage symptoms of various mental health problems and conditions such as depression, anxiety, substance abuse, posttraumatic stress, and eating disorders [49,50]. Thus, incorporating mHealth apps into mental health treatment and adjunctive interventions may prove beneficial.

Furthermore, incorporating mHealth apps into established evidence-based interventions may also serve as a culturally inclusive way of disseminating treatment to younger, more technologically savvy generations who also happen to demonstrate higher rates of suicidal thoughts and behaviors than adults [6]. mHealth apps may also help address service use barriers and risk factors (eg, stigma) that hinder individuals from seeking help and participating in treatment for suicidality. Combining suicide safety planning practices with mHealth apps may combat accessibility concerns as well, including a commonly reported flaw of the traditional intervention—the reliance on a paper format [35]. Given the widespread proliferation of mHealth apps for suicide prevention, there is a need to examine the components and features that have been incorporated into the design of suicide safety planning apps.

### Objectives

The purpose of this systematic literature review was to first assess the extent to which suicide safety planning mHealth apps integrated the 6 steps or components of a widely used safety planning intervention (SPI) developed by Stanley and Brown [31] (research question [RQ] 1). Next, we independently reviewed available mHealth suicide safety planning apps via download from iOS and Android app stores to assess the integration of SPI components and to categorize different app design features used to personalize the end users’ experience (RQ2). We also examined the evidence on the effectiveness of these apps in terms of usability, acceptability, app engagement, and suicide-related outcomes (RQ3). This review aims to synthesize the extant research to inform suicide prevention efforts, clinical practice, and future development of suicide safety planning mHealth apps.

## Methods

### Overview

In accordance with the PRISMA (Preferred Reporting Items for Systematic Reviews and Meta-Analyses) 2020 statement guidelines (Multimedia Appendix 1 [51]), a comprehensive systematic review of existing literature on suicide safety planning via mHealth apps was conducted. The process is described in the following sections.

### Systematic Literature Review

#### Eligibility Criteria

The inclusion criteria for the reviewed research studies were as follows: (1) a primary focus on suicide safety planning involving the use of a mHealth app, (2) publication in a peer-reviewed article written in English, and (3) availability of the full text of the article. Studies were excluded if (1) the word suicide, safety plan, or app was not included in the title; (2) they included other forms of mHealth technologies as the primary focus (eg, web-based applications); (3) the apps were designed with safety planning as a secondary focus (ie, not exclusively for suicide safety planning, not intended as a crisis intervention, or use of safety planning as a secondary tool to other treatment modalities); and (4) they were part of other systematic reviews or meta-analyses. We included studies across the entire system development life cycle (eg, formative evaluations and 1 group pre-posttest designs) owing to limited research on the topic and the relatively recent emergence of such research.

#### Information Sources

The following 5 bibliographic databases were used to systematically review the literature: PsycINFO, PubMed, ACM Digital Libraries, Academic Search Premier, and ERIC. We limited our results to articles published between January 2000 and May 2023. All databases were last searched on July 2, 2023.

#### Search Strategy

We used the following keywords to search for the topic of interest in each scientific database: “Safety Plan*” AND (“Applications” OR “Apps”); (“Suicide” OR “Safety Plan*”) AND (“Applications” OR “Apps”); “Suicide Interven*” AND (“Applications” OR “Apps”); “Suicide Prevent*” AND (“Applications” OR “Apps”); “Suicide Contract” AND (“Applications” OR “Apps”); “mHealth” AND “Suicide”; “Crisis Response” AND “Plan*.” Asterisks were added to search for words that began with the preceding letters (eg, prevent*: prevent, prevention, and preventing). An example of the search strategy outlined above is provided in Multimedia Appendix 2.

#### Selection Process

**Figure 1:**
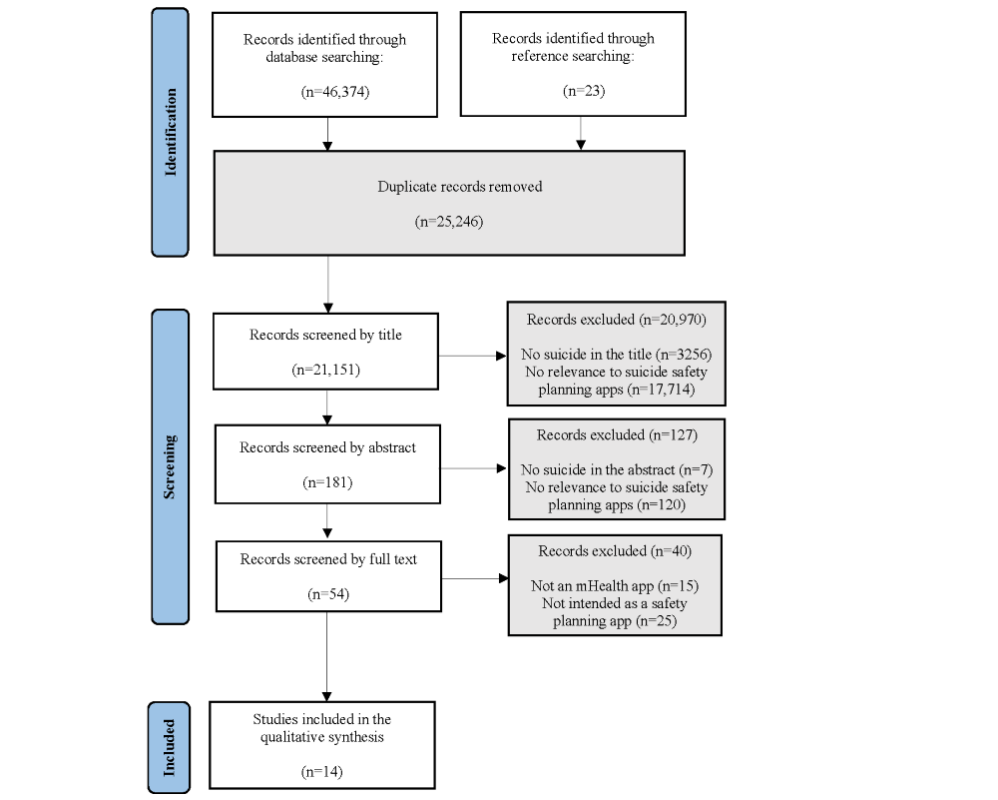
Flowchart of the studies in line with the PRISMA (Preferred Reporting Items for Systematic Reviews and Meta-Analyses) guidelines. mHealth: mobile health.

Citations obtained from electronic databases were imported into Zotero (version 6.0.16). Two reviewers (KG and VLO) independently screened the articles to remove duplicates and assessed inclusion and exclusion criteria by title and abstract. For articles about which the reviewers were uncertain after the title and abstract review, 4 reviewers independently analyzed the full-text articles to determine whether they met the inclusion criteria. The reviewers discussed discrepancies until they reached a consensus. The references of all articles that met the inclusion criteria were reviewed and cross-referenced for additional relevant articles. We included all eligible studies (N=14) in this systematic review (Figure 1 [51]).

#### Data Collection Process

Data from eligible studies were analyzed using the Cochrane Collaboration’s data extraction template for included studies (version 1.8) [52]. We added study-specific items to the template to answer RQ1 and RQ2. Specifically, to answer RQ1, we reviewed articles describing each mHealth app and coded, using a dichotomous (yes or no) coding scheme, for the following SPI components: (1) personal warning signs, (2) coping strategies, (3) ways to distract oneself through social activities, (4) identification of and ways to access trusted individuals (eg, family and friends) for support, (5) identification of and ways to access community supports (eg, mental health professionals, nonmental health adult supports, crisis, or emergency services), and (6) information about keeping the environment safe (eg, restricting access to lethal means). To answer RQ2, we downloaded available mHealth apps via the Apple App Store or Google Play Store or contacted app developers to conduct an independent review of SPI components and app features described in the articles. Next, we created codes to describe app features, organized and categorized codes based on similarities, and generated 5 themes to capture the core aspects of features. To answer RQ3, we extracted both qualitative and quantitative findings reported on primary and secondary outcomes. We categorized the study outcomes into 3 main research themes.

Two reviewers coded 2 research articles to assess interrater reliability based on the coding template and made refinements as necessary (eg, added operational definitions to describe SPI components and provided examples of app features). Once finalized, the reviewers used the template to extract the data from the remaining studies. Data items included (1) general article information (eg, author, publication year, and country); (2) study methods (eg, aims and research design); (3) study characteristics (eg, sample size, sample demographics, and setting); (4) SPI intervention characteristics (RQ1); (5) mHealth app design features (RQ2) and primary and secondary outcomes (RQ3); and (6) study implications and future directions (Multimedia Appendix 3 [42,43,53-64]). A similar process was used to independently code the SPI components and app features of the mHealth apps available for download.

#### Risk of Bias Assessment

The risk of bias for each study was assessed by 2 independent reviewers (KG and VLO) using Joanna Briggs Institute (JBI) appraisal tools for quasi-experimental [65] and qualitative research study designs [66]. For studies that included mixed methods designs, we used both tools as recommended by the JBI. Each appraisal tool used a rating scale with yes, no, unclear, and nonapplicable responses. The overall appraisal rating was based on the following categories: include, exclude, and seek further information. Disagreements between the reviewers were discussed until they reached a consensus (Multimedia Appendix 4 [65,66]).

#### Synthesis of Results

Owing to the heterogeneity of the study designs, participants, and outcomes collected, we could not perform a meta-analysis of the identified studies in this review. Therefore, we present a narrative synthesis of the study findings.

## Results

### Study Selection

The initial search of electronic databases and hand-searched references resulted in a total of 46,397 peer-reviewed articles. After duplicate records were removed, 21,151 studies remained. Titles were screened for relevancy (eg, relating to suicide, suicide safety planning, and mHealth apps), and 20,970 articles were excluded. A total of 181 abstracts were reviewed. Following full-text reviews of 54 articles, 40 articles were removed (15 studies did not include an mHealth app and 25 were not intended as a suicide safety planning app). A total of 14 articles met the inclusion criteria (refer to Figure 1 for breakdown).

### Study Characteristics

#### Overview

The detailed study characteristics of the selected articles (N=14) are presented in Multimedia Appendix 3. Most studies (12/14, 86%) were conducted outside the United States [42,53-63]. The year range of the selected articles was between 2015 and 2023.

#### Study Design

As shown in Tables 1 and 2, a total of 7 mHealth suicide safety planning apps were studied across the 14 articles in our data set (Multimedia Appendix 3). We classified the articles based on the research design (ie, formative feedback, usability assessment, single cohort pre-posttest, and random control trial protocol). Formative designs assessed SPI components and features to guide app development [43,56,61,64], whereas usability designs assessed interface design issues and functionality (eg, task difficulty and time to complete tasks) [55,60,61,64]. Other studies evaluated the acceptability or feasibility of a fully developed mHealth app [54,58-60,62,63]. Across these studies, participants rated the frequency and duration of app use; ease of navigation; and level of satisfaction, comfort, confidence, or engagement in using the app.

**Table 1:** Formative, usability, and acceptability assessments of mobile health suicide safety planning apps (n=8 articles).

Safety planning app	Year span	Formative feedback only	Usability assessment	Random control trial	Use period	Key findings
Unnamed [43]	2015	Clinicians (n=9), at-risk youth, and guardians (n=20)	N/A^a^	N/A	None	Qualitative feedback to inform app design; no app developed
SERO [56]	2022	Clinicians, at-risk individuals (n=11), and relatives (n not reported)	N/A	N/A	None	Summary of 6 suicide prevention strategies aligned with app design
ED-SAFE [64]	2023	Clinicians and subject matter experts (n=7), and at-risk adults (n=6)	Emergency department patients after discharge (N=14)	N/A	Unclear	High usability scores and low uptake (use); no significant clinical outcomes reported
MYPLAN [53,54,62]	2016, 2017, and 2020	At-risk youth, adults, relatives, and clinicians (n=26)	N/A	Protocol only (N=546)	Familiarity with app	Qualitative feedback only; no clinical outcomes assessed
SafePlan [57,61]	2020 and 2023	Clinicians and experts (N=15)	Students (N=18)	Protocol only (N=80)	Single use	High usability scores; no clinical outcomes assessed

^a^N/A: not applicable.

**Table 2:** Pre-posttest assessments of mobile health suicide safety planning apps (n=6 articles).

Safety planning app name	Year span	Formative feedback only	Usability assessment	Single cohort pre-posttest	Use period	Key findings
Brake of My Mind [55]	2020	Expert heuristic evaluations (N=5)	Clinician interviews (N=6)	At-risk youth (N=3)	1 week	High usability scores; sample size too low to assess clinical efficacy
BackUp [42,60,63]	2017, 2018, and 2022	Designed informed by the expert panel (n=8) and at-risk adults (n=21)	Usability assessment combined with pre-posttests	Protocol only, single cohort design (N=80); at-risk adults (n=21) and at-risk adults (n=12)	1 week and 3 months	High usability scores, no significant decrease in suicidal thoughts; low clinical and patient uptake (use)
BeyondNow [58,59]	2019 and 2020	N/A^a^	N/A	At-risk youth and adults (n=22) as well as at-risk youth (n=17)	2 months and 6 weeks	High usability scores. Significant reduction in severity and intensity of suicidal ideation; significant increase in coping; and no significant change in suicide resilience for mixed samples. For youth only sample, no significant decrease in suicidal thoughts and significant increase in suicidal resilience. No conclusions regarding clinical efficacy.

^a^N/A: not applicable.

#### Sample Characteristics

Across studies, the study sample varied in age, type of participant (eg, youth or adults at risk of suicide and clinicians collaborating with suicidal clients), and setting (eg, suicide prevention clinic and pediatric inpatient facility). Among studies that recruited participants to inform or evaluate mHealth suicide safety planning apps [43,54-56,58-64], the sample size ranged from 11 to 36 participants. However, after reporting dropout rates, sample sizes dropped to as low as 2 participants and as high as 22 participants.

### Integration of SPI Components Within mHealth Apps

Most articles (5/7, 71%) describing the mHealth apps incorporated SPI components into the design of their apps [54,58,61,63,64] (Table 3). Creating a safe environment from lethal means was the missing component in 29% (2/7) of the apps [55,56].

**Table 3:** Safety planning intervention (SPI) components and app features.

		MYPLAN (MinPlan) [54,62]	BoMM^a^ [55]	BeyondNow [58,59]	SafePlan [61]	BackUp [63]	ED-SAFE [64]	SERO [56]
**SPC^b^**								
	SPI 1: warning signs	X^c^	X	X	X	X	X	X
	SPI 2: coping strategies	X	X	X	X	X	X	X
	SPI 3: distractions social activities	X	X	X	X	X	X	X
	SPI 4: trusted supports (family and friends)	X	X	X	X	X	X	X
	SPI 5: community supports (MHP^d^)	X	X	X	X	X	X	X
	SPI 6: safe environment (lethal means)	X	—^e^	X	X	X	X	—
**Interactive features^f^**								
	Links stressors to cope strategies; SPI 1 and 2	X	—	—	X	—	—	X
	Inclusion of media (distraction); SPI 3	X	X	✓^g^	X	X	X	—
	Access to trusted supports; SPI 4	X	X	X	X	X	X	X
	Access to community supports; SPI 5	X	X	X	X	X	X	X
	GPS tracking; SPI 5 and 6	X	X	—	✓	—	—	—
**Individualized user experience**								
	Revisable safety plan	X	X	X	X	X	X	X
	Personality and mood exercises or tracking	—	X	—	X	—	—	X
	Visual customization	✓	X	✓	X	X	—	X
	Enabling notifications	X	—	—	X	✓	—	✓
**Interface design**								
	Easy to navigate	X	X	X	X	X	X	✓
**Guidance and training**								
	In-app tutorial	X	X	✓	—	X	X	✓
**Privacy and sharing**								
	Secure username and password	✓	X	—	X	✓	X	✓
	Shareable data and safety plan	X	—	X	X	—	X	✓

^a^BoMM: Brake of My Mind.

^b^SPC: safety planning component.

^c^SPC or app feature included in the app.

^d^MHP: mental health professional.

^e^SPC or app feature missing in the app.

^f^Denotes innovative app features aligned with SPI components.

^g^Feature included in the app that was not mentioned in the article.

We used the JBI quasi-experimental appraisal tool [65] to assess the risk of bias across 5 studies [55,58-60,63]. These studies did not include a control or comparison group, increasing the threat to internal validity. Pre- and posttest measures were used to assess the immediate effects of the mHealth apps. However, the lack of repeated outcome measures over time, selection bias (nonrandom samples), and small sample sizes pose a risk of bias within and across these studies.

The qualitative appraisal checklist tool [66] was used to assess the risk of bias in 4 studies [43,54,56,62]. Across 2 studies [43,54], the cultural or theoretical orientation of the researchers and their influence on the research process was unclear. These issues were noted in the other 2 studies [56,62] as well. In these studies [56,62], it was also difficult to identify the philosophical perspective and congruity between the research methods, data analysis, and interpretation. The studies included more of a description of the design of the apps and included general perceptions from stakeholders.

The remaining studies [61,64] were assessed using both the quasi-experimental and qualitative appraisal tools owing to their mixed methods designs. In both studies, it was unclear whether the researchers’ cultural or theoretical orientation, their influence on the research, and the adequate representation of the participants and their voices were addressed. Other key issues included the lack of a control or comparison group, nonrandom and small sample sizes, and the use of posttest measures to assess usability at only 1 time point. JBI appraisal results are included in Multimedia Appendix 4.

On the basis of our independent review of available mHealth suicide safety planning apps, SPI components described in each article were verified in 71% (5/7) of the apps [54,56,58,61,63]. The app features described in the articles were also confirmed in these apps. App features not highlighted in the articles but found within the apps are listed in Table 3. We were unable to verify SPI components and app features in 2 of the reviewed apps in the literature [55,64].

### Comparative Analysis of SPI Components and App Features

In our analysis of the literature and available mHealth apps for download, we synthesized the commonalities of app features and categorized them into 5 broad themes: interactive features, individualized user experience, interface design, guidance and training, and privacy and sharing. These features are described in the following sections.

#### Interactive Features

Three of the suicide safety planning mHealth apps [54,56,61] allowed users to associate suicide warning signs or precipitating stressors with their personalized coping strategies (aligns with SPI 1 and 2 in Table 3). O’Grady et al [61] stressed the importance of including this feature in apps, as this functionality can serve to preemptively address an impending crisis before it fully manifests. Most of the suicide safety planning mHealth apps (6/7, 86%) also included social distractor features in which users had access to their phone’s camera with the ability to upload or view media content (eg, pictures, quotes, music, activities, videos, and inspirational stories; SPI 3) [54,55,58,61,63,64]. In the *BackUp* app [63], loved ones, trusted supports, and suicidal users were able to upload media and share content to inspire hope and distract users from negative thinking.

Each mHealth app also included a built-in feature for users to save and contact trusted individuals within their social support networks (SPI 4). Typically, users entered contact information into the mHealth app directly or linked to their contact directories. A unique feature of the *MYPLAN* app [54] allowed users to create prewritten messages that they could send to their social supports during times of distress. Although this feature was created to inform loved ones of the app user’s emotional state during a crisis, participants (ie, app users) noted concerns about messages being misunderstood, whereas relatives felt that messages could minimize emotional states or provide inaccurate information about the app user’s safety. All apps included the ability to access community supports such as mental health professionals (SPI 5). Three apps [54,55,61] included GPS capabilities, which enabled users to search for nearby counseling agencies or emergency services, and, after selecting a search result, users received directions for quick access (SPI 5 and 6). The *ED-SAFE* app [64] included a referral search engine that allowed users to find behavioral health care by specialty and zip code. Emergency service numbers, mostly displayed via a phone icon or brief words (eg, “Crisis”), were clearly visible (listed on all pages) in 57% (4/7) of the mHealth apps [56,58,61,63], which is the suggested ethical guideline from prior work [67]. Three apps did not include access to emergency service numbers on all pages but provided them somewhere else within the app [54,55,64].

#### Individualized User Experience

All apps (7/7, 100%) allowed users to continually add to or revise their safety plans. Examples included the addition of new warning signs, reasons for living, and identifying coping strategies. None of the apps maintained a historical record of the previous safety plans or provided a visual mechanism to track daily, weekly, or monthly patterns based on stressors encountered or coping strategies used. Other personalization aspects included the ability to enable or disable therapeutic modalities [61], the inclusion of web-based resources to take an aptitude and personality test [55], exercises to express moods [55], and mood tracking [55,56,61]. In addition, all apps had built-in features to make esthetic customizations, such as personalizing the home screen, changing the color palate, and adding background pictures [54-56,58,61,63]. In 57% (4/7) of the apps, notifications were enabled to remind users about using their safety plan or skills to practice [54,56,61,63].

#### Interface Design

Several studies used iterative feedback from content and app design experts to create easy-to-navigate interfaces [58,61,63]. To enhance the navigation experience, a simple layout, clear or user-friendly language, and accessibility features were important design considerations included in some mHealth apps [54,58,61,64]. For example, *SafePlan*’s layout mimicked the paper version of the safety plan to better transition users from using the paper version to the app [61].

#### Guidance and Training

In-app tutorials or instructional videos were included in 86% (6/7) of the suicide safety planning mHealth apps [54-56,58,63,64]. Some of these tutorials focused on how to use the app, whereas others explained the safety planning process. For example, the *BeyondNow* app [58] included a video outlining the process of safety planning and links to other helpful information. The most extensive tutorials were seen in the companion app to *ED-SAFE* [64], where tutorials could be received from a female provider, a male community member, or an avatar. The mHealth suite of apps also included self-care education materials about suicidality, safety plans, and life plans. In addition, the *BackUp* app [63] provided supportive contacts with web-based information on ways to identify warning signs and strategies to talk with suicidal individuals. The *Brake of My Mind* app [55] included an introduction from the developer with additional web-based resources to increase app usability.

#### Privacy and Sharing

Researchers also highlighted app privacy and sharing capabilities as important features to consider when designing mHealth suicide safety planning apps. Given the personal nature of the information saved, most mHealth apps required a username and password to log in [54-56,61,63,64]. For example, *ED-SAFE* [64] used the username and password feature to verify user identity and connect information collected in the emergency department setting to the mHealth app. Other apps disabled GPS for location tracking or did not use external servers to store users’ information for privacy and security concerns [61,63]. Several apps (5/7, 71%) included features allowing users to share self-monitoring data or share safety plans with clinicians or trusted individuals [54,56,58,61,64]. For instance, *ED-SAFE* [64] allowed users to share safety plans as well as appointment information, self-care education, helplines, referrals, and distractions through password-protected privileges given to authorized family members.

### mHealth App Evidence of Effectiveness

The qualitative and quantitative findings were categorized into 3 main research themes: app usability and acceptability, app use and engagement, and suicide-related outcomes.

#### App Usability and Acceptability Findings

Across 71% (10/14) of the studies [54-56,58-64] that assessed the initial usability or acceptability of mHealth suicide safety planning apps, stakeholders’ experiences testing the mHealth apps were generally positive. Four studies [55,60,61,64] included standard rating scales (ie, System Usability Scale [68]) to assess the perceived usability of their apps, and scores exceeded the minimum usability standards (ie, >70). The remaining studies used qualitative feedback from focus groups, case reports, and open-ended questionnaires. For example, in the study by Buus et al [54], participants found the *MYPLAN* safety planning app useful in recognizing patterns of impending crises and for reinforcing personalized strategies to cope with distress. In describing the benefits of the *BeyondNow* safety planning app, participants in the study by Melvin et al [58] reported developing a sense of hope and connection from using the app. Researchers have attributed these findings to the accessibility of the app and its customizable features. According to the authors, stakeholders regarded apps as highly intuitive, easy to use, and visually appealing interface in terms of the design [59,61,62,64].

#### App Use and App Engagement

Five studies examined app use over time [58-60,63,64]. Overall app engagement and use were minimal. Across 3 studies, >70% of the participants used the apps at least once during the testing period, which ranged from 1 to 10 weeks [58,59,63]. In the study by Melvin et al [58], 77% (17/22) of the participants reported using the mHealth app “occasionally” or “a lot,” including to make changes to safety plans. Most participants also reported using the mHealth app during a suicidal crisis (15/22, 68%) or when experiencing suicidal ideation (18/22, 82%). Increased frequency of app use during a crisis or among participants with high levels of suicide ideation was reported in studies by Pauwels et al [63] and Muscara et al [59]. Larkin et al [64] reported that 2 (40%) out of 5 participants reported downloading the *ED-SAFE* patient mHealth app after discharge. Low uptake rates were mostly attributed to the participants’ forgetfulness to download the app. Although most participants acknowledged the benefits of using mHealth suicide safety planning apps during times of crisis [58,63], participant feedback from the study by Muscara et al [59] suggested that participants did not believe or were unsure whether the use of the *BeyondNow* safety planning app could help them manage their symptoms or keep individuals safe during a crisis. Only 35% (6/17) of the participants favored using the app in the future. Conversely, participants in the study by Nuij et al [60] noted that easy access to the *Backup* mHealth app provided a sense of reassurance and helped to deter suicidal thoughts.

#### Suicide-Related Outcomes

Suicide-related outcomes were examined across 29% (4/14) of the small-scale pilot studies (with sample sizes ranging between 3 and 22) [55,58,59,63]. The study by Jeong et al [55] assessed the Theory of Planned Behavior constructs, including attitudes, subjective norms, perceived behavioral control, and intentions toward engaging in suicide attempts, using a pre-posttest design with a small (N=3) sample of adolescent survivors of suicide attempts. The results showed statistically significant changes in attitudes, perceived behavioral control, and intentions, suggesting that the suicide safety planning app helped to positively shift attitudes toward life and reduce beliefs and intentions to engage in self-harm behavior.

Suicide coping or resilience was evaluated in 2 studies using pre-posttest designs [58,59]. Both studies used the same safety planning app (ie, *BeyondNow*) to examine the changes in protective factors. Melvin et al [58] found a statistically significant increase in suicide-related coping among youth and adult participants (n=22). This finding suggests an increase in knowledge and confidence to use internal coping strategies and external resources to manage suicide ideation. However, the researchers did not observe statistically significant changes in suicide resilience (ie, the perceived ability to manage suicidal thoughts and feelings). In contrast, Muscara et al [59] found a significant increase in 1 subscale of suicide resilience, emotional stability (ie, the ability to regulate emotions), among youth participants (N=17) in their study.

Suicidal ideation or self-harm behavior were measured in 3 studies [58,59,63]. In an open-label, single-group design, Melvin et al [58] found statistically significant reductions in both the severity and intensity of suicide ideation following exposure to an 8-week trial that evaluated the clinical effectiveness of using the *BeyondNow* suicide safety planning app as an adjunct to treatment as usual (ie, existing mental health services). In an evaluation of the same mHealth app, but with the addition of a personalized toolbox app (ie, *BlueIce*), instead of treatment as usual, Muscara et al [59] also found a reduction in suicide ideation and self-harm behaviors (ie, attempts to harm oneself with and without suicidal intent). However, these findings were not conclusive or statistically significant owing to the small sample size and lack of a control group. Pauwels et al [63] found a similar, nonsignificant decrease in suicide ideation scores in a study examining pre-posttest changes following exposure to the *BackUp* suicide safety planning app. Although these studies provide some evidence of clinical utility, these researchers noted study limitations and the need for further evaluation using randomized controlled trials (RCTs).

## Discussion

### Principal Findings

The primary aim of this study was to conduct a comprehensive analysis of the integration and inclusion of the SPI components developed by Stanley and Brown [31] in the design of mHealth suicide safety planning apps. The secondary aim was to synthesize and assess the research methods of studies that reported on the effectiveness of these apps. Implications of these findings and practical recommendations for future directions in mHealth suicide safety planning research are described in the following sections.

### Integrating Components of Suicide Safety Planning Into mHealth Apps

Overall, most apps included the core components of the SPI developed by Stanley and Brown [31], such as the identification of suicide warning signs, coping strategies, and supportive persons. Therefore, the results from this review provide evidence of some level of successful integration of SPI components into mHealth suicide safety planning apps (RQ1). Lethal means safety was 1 component that was not incorporated in 2 of the apps reviewed. Reducing access to lethal means is a critical part of suicide safety planning [31] and warrants inclusion in mHealth apps as it brings attention to methods that could be used to attempt or die by suicide if not removed from a user’s environment.

An important aspect of suicide safety planning is access to one’s safety plan. In this review, having access to safety plans at any time [54,55,58,60,61] and being able to continually revise the plan were considered benefits over traditional paper-based safety planning. In some apps, users could create associations between different suicide safety planning components (SPCs; eg, triggers and coping strategies) to better contextualize their experiences and create actional plans for mitigating crises [54,56,61]. We recommend that additional linkages between the SPCs be included to further personalize users’ experiences.

Despite the integration of SPI components within mHealth suicide safety planning app designs, we also identified important gaps in the literature that warrant the attention of app designers, researchers, and mental health professionals who may use this type of technology within their clinical practice. For instance, researchers have consistently emphasized the importance of completing the initial safety plan alongside a knowledgeable clinician [42,54,58,61] to ensure that at-risk users and loved ones understand the components and purpose of a safety plan. However, many of the analyzed apps allowed users to complete the safety plan without the recommended clinical support, and in some cases, they lacked disclaimers. Therefore, additional guidance from a professional when using mHealth suicide safety planning apps would further serve to assist users and ensure that the safety planning process is carried out as intended.

This review also found that most of the apps did not go beyond the traditional SPCs of paper-based protocols to integrate more interactive features that could potentially improve adherence or engagement. For instance, daily or weekly check-ins have been shown to improve adherence in other mHealth contexts, such as for smoking cessation [69] and the management of schizophrenia [70]. Visualization graphs of patterns or trends in suicide warning signs, triggers, and coping behaviors logged over time may serve to increase engagement and improve outcomes, as visualizing behavior change over time has been recommended in other mHealth contexts [71], such as alcohol reduction [72]. Furthermore, other meaningful ways to actively and continuously engage one’s support contacts (eg, clinicians, parents, and family members) and to reinforce the use of healthful coping strategies would be an advantageous direction for future exploration in mHealth app design. Beyond general support contacts, prior research has found that parental support is a significant protective factor against youth suicide [73,74]. For youth, in particular, it may be advantageous to include parents, family members, or other trusted adults in the mHealth suicide safety planning process to increase uptake, enhance help-seeking and coping behaviors, and reinforce ways to keep one’s environment safe. However, future research would need to carefully design and evaluate such interventions to ensure they are effective before making these interventions widely available through the dissemination of mHealth apps for suicide safety planning.

Another variation across the apps was that some apps provided default values for suicide SPCs (eg, suggested coping strategies), whereas others did not. Therefore, an area of future research could be to study whether providing default values is beneficial or detrimental to the safety planning process. Finally, rather than training focused on the technical aspects of using the mHealth app, there is a need to include psychoeducation for suicide safety planning [75], especially related to coping strategies and lethal means restriction, which should be modeled as a collaborative process between at-risk users and their support systems [76].

### Usability and Design Considerations for mHealth Suicide Safety Planning Apps

Overall, our review highlights three important recommendations to consider when designing safety planning mHealth apps (RQ2): the need to (1) encourage end user collaboration in the design and implementation of the intervention, (2) incorporate personalization or customization capabilities, and (3) develop appropriate privacy safeguards to prevent liability and address other safety concerns that may arise when integrating mental health care and technology. A key strength of most studies in our review was the interdisciplinary collaboration between app developers, computer scientists, and clinical researchers that facilitated the design, development, and evaluation of the various mHealth suicide safety planning apps. In addition, multiple stakeholders were included in the design process, including individuals at risk of suicide, clinicians, usability experts, parents, and extended family members. Only in 1 instance, end users engaged who were not considered part of the target population of at-risk users (eg, students). We strongly recommend that future research continue to include researchers from across multiple disciplines (eg, psychology, public health, social work, medicine, computer science, and human-computer interaction), intended end users, and mental health professionals across each stage of the research process. For instance, researchers from different disciplines may be able to raise important threats to validity during the research design process that could lead to more robust study designs.

A key weakness highlighted within several studies was limited uptake or sustained use of the mHealth suicide safety planning apps over time. Such findings shed suspicion on the feasibility of this type of intervention being effective outside of research, regardless of the high usability and acceptability ratings. Some studies attributed lack of use to the reduction of suicidal behaviors over time, but others suggested that the suicide safety planning process, as designed to be carried out within the apps, was only suited for in-crisis situations and not appropriate for sustained use over time. Although this may be the case, it is also possible that the lack of interactive or engaging features within the apps made them less appealing to users. Being able to customize and personalize app features may help to enhance the user’s experience and increase app engagement. Many of the apps included social distractions (ie, music and pictures) or other features, such as diary cards, which might help increase overall app engagement during noncrisis periods. However, as suicidality is episodic, future research should be conducted to understand how different modalities or features (eg, mood tracking, journaling, mindfulness, and art) could be combined with suicide safety planning in a complementary way for long-term use and engagement. Future work should also consider leveraging advanced technologies and assessments, such as artificial intelligence and ecological momentary assessments [77,78], that could be used to anticipate heightened suicide risk and prompt users to engage in the mHealth app suicide safety planning process when they need it most.

### Threats to Validity and Inconclusive Clinical Outcomes Associated With the Use of mHealth Suicide Safety Planning Apps

This review provides some preliminary evidence suggesting that suicide safety planning via mHealth apps could be an easy-to-use mechanism to provide individualized care to those who may otherwise go unserved due to common treatment barriers (RQ3), such as poor accessibility to service providers, lack of knowledge about suicide, and stigmatizing beliefs about help seeking [20-24]. At the same time, several threats to validity were uncovered by our assessment of risk bias, which can inform directions for future research. First, the robustness of the qualitative studies could be improved by stating the positionality of the researchers as well as a clear justification for the design of the mHealth apps. In some cases, articles were published by interdisciplinary teams, whereas in other cases, authors appeared to be from a single discipline (eg, computer science). Details about the composition and expertise of the research team are important, as well-implemented mHealth apps require interdisciplinary skill sets that span clinical, design-based, and technical expertise. Furthermore, the quantitative studies analyzed in our review were constrained by small sample sizes and no published RCTs. Among the pre-posttest studies conducted thus far, the clinical outcomes were inconclusive.

As such, RCTs with control groups, random assignment, and repeated measure outcomes assessed over time are needed in the future to evaluate the efficacy of using suicide safety planning mHealth apps compared with traditional paper-based safety plans [54,57], specifically related to reducing suicidal urges and behaviors and increasing use of coping strategies, as well as increased engagement in crisis and mental health services after the crisis. When doing so, researchers should recruit larger samples to ensure that the results are conclusive and can be generalized to the populations of interest. Furthermore, additional use metrics collected by the apps to track behavioral data associated with using different app features, such as user engagement with the 6 components of the SPI developed by Stanley and Brown [31], should be considered to better understand the potential mediating factors and behaviors that may influence clinical outcomes. Although the usability of the apps would be an important consideration to control for in future studies, it is necessary to move beyond such measures to determine the efficacy of mHealth apps in reducing suicide-related outcomes. In summary, the inclusion of more advanced study design methodologies and recommendations from lessons learned in future mHealth apps could serve to mitigate suicide risk and promote overall safety.

### Limitations and Future Research

This systematic review included 14 peer-reviewed articles that designed, developed, and evaluated mHealth apps for suicide safety planning. There are several limitations of this study that should be addressed in future research. First, although our search process was comprehensive, it is possible that our keywords missed relevant articles and mHealth apps that should have been included in the review. Second, as many of the apps described in the articles were not publicly available for download, we requested access from the corresponding authors to conduct our review. In 2 cases, we were unable to gain access to the apps; therefore, our analysis was based on the description of those apps based on the published paper. As such, it may be possible that some features were not described in the original papers; thus, they were not included in our review. Future research should also consider conducting a systematic feature analysis of mHealth suicide safety planning apps that are publicly available for download but not studied within the peer-reviewed literature. Finally, a limited number of published RCTs at the time of the review restricted our ability to report on app use and suicide-related outcomes. As such, the main call-to-action from this review is the need to move beyond usability studies of newly developed mHealth suicide safety planning apps to robust clinical research designs to examine their efficacy in reducing suicidality among at-risk user populations.

### Conclusions

Overall, most articles included in this review did little to evaluate the efficacy of mHealth suicide safety planning apps beyond usability assessments, signaling that these apps and corresponding research are still in their infancy in terms of validating clinical outcomes. Although most of the mHealth safety planning apps included in our review are not yet downloadable and broadly available for public use, the prevalence and popularity of mHealth suicide prevention and mental health support apps on the open market that have been deployed without rigorous peer-reviewed research is a concern. As such, there is a critical need for future research to ensure that mHealth apps for suicide safety planning integrate the lessons learned from empirical user-based and clinical research, are upheld to high ethical mental health care standards, and show clinical efficacy for reducing suicidality before the apps are released to end users. This is especially true given the delicate and important goal of preventing suicide among at-risk populations. It is promising to see that future randomized clinical trials have been registered to build upon this important preliminary work on mHealth suicide safety planning apps.

## Appendix

Multimedia Appendix 1 PRISMA (Preferred Reporting Items for Systematic Reviews and Meta-Analyses) checklist.

Multimedia Appendix 2 Example search strategy.

Multimedia Appendix 3 Detailed summary of the selected articles and key findings (N=14).

Multimedia Appendix 4 Critical appraisal results.

## Abbreviations

JBI: Joanna Briggs Institute
mHealth: mobile health
PRISMA: Preferred Reporting Items for Systematic Reviews and Meta-Analyses
RCT: randomized controlled trial
RQ: research question
SPC: safety planning component
SPI: safety planning intervention
