# Supplementary material for: Translating Suicide Safety Planning Components Into the Design of mHealth App Features: Systematic Review
Source: JMIR Ment Health. 2024 Mar 28;11:e52763. doi: 10.2196/52763 (PMC11009854; doi:10.2196/52763)
Supplement: Multimedia Appendix 2 [file mental_v11i1e52763_app2.docx]

**Appendix 1.** Example Search Strategy.

| **Selected Field** | Title |  | |  |  |  |  |  |
| --- | --- | --- | --- | --- | --- | --- | --- | --- |
| **Publication Year** | 2000-2023 |  |  |  |  |  |  |  |
| **Search Modes** | find all my search terms |  |  |  |  |  |  |  |
| **Limit your results** | *Linked Full Text *Open Access *Scholarly (Peer Reviewed) Journals |  |  |  |  |  |  |  |
| **Publication Status** | All | **Publication Type** | Peer Reviewed Journal |  |  |  |  |  |
| **Language** | English | **Document Type** | Journal Article |  |  |  |  |  |
| **Age Groups** | All |  | |  |  |  |  |  |
| **Intended Audience** | All |  |  |  |  |  |  |  |
| **Key Terms for Searches** | | | |  |  |  |  |  |
| Search 1 | “Safety Plan*” AND (“Applications” OR “Apps”) | | |  |  |  |  |  |
| Search 2 | (“Suicide” OR “Safety Plan*”) AND (“Applications” OR “Apps”) | | |  |  |  |  |  |
| Search 3 | “Suicide Interven*” AND (“Applications” OR “Apps”) | | |  |  |  |  |  |
| Search 4 | “Suicide Prevent*” AND (“Applications” OR “Apps”’) | | |  |  |  |  |  |
| Search 5 | “Suicide Contract” AND (“Applications” OR “Apps”) | | |  |  |  |  |  |
| Search 6 | "mHealth" AND "Suicide" | | |  |  |  |  |  |
| Search 7 | "Crisis Response" AND "Plan*" | | |  |  |  |  |  |
|  |  |  |  |  |  |  |  |  |
