# Supplementary material for: Translating Suicide Safety Planning Components Into the Design of mHealth App Features: Systematic Review
Source: JMIR Ment Health. 2024 Mar 28;11:e52763. doi: 10.2196/52763 (PMC11009854; doi:10.2196/52763)
Supplement: Multimedia Appendix 3 [file mental_v11i1e52763_app3.docx]

**Appendix 2.** Detailed Summary of Selected Articles and Key Findings (*n*=14).

| **Author, Year** | **Country Origin** | **Research Design/Study Objective** | **Sample/Targeted Characteristics** | **Key Findings/Future Directions** |
| --- | --- | --- | --- | --- |
| Andreasson et al., 2017, *MYPLAN* [55] | Denmark | **Research Design:** (1) RCT Research Protocol Only  Developed mHealth app (MYPLAN) study protocol for **future** randomized control trial (RCT) to assess suicidal ideation, hopelessness, depression, and user satisfaction compared to Treatment as Usual (TAU, written safety plan) condition | **Targeted Sample** (**future**): (1) youth and adult clients (no age restriction) at risk of suicide receiving short-term therapy (n=546 participants; n=273 each arm) | ***Key Findings:*** Not applicable. Future work.  ***Considerations:***  -Smartphone features could increase the effectiveness of a crisis plan (communication tools, GPS, self-assessment tools)  -Access/availability of mHealth apps may be helpful for fluctuating nature of suicide ideation (which could be monitored over time)  ***Future Directions*:**  -Future pilot study to examine technical issues and feasibility before conducting a RCT |
| Buus et al., 2020, *MYPLAN* [56] | Denmark | **Research Design:** (1) Acceptability  Examined stakeholders’ expectations and experiences using MYPLAN (mHealth safety planning app) | **Study Sample:** (1) focus groups conducted with stakeholders from suicide prevention clinics who were MYPLAN users. User engagement levels with the app were not reported.  Stakeholders included n=5 youth and n=8 adult clients (ages 14-28) who received clinical treatment for suicide risk and/or other psychosocial problems, n=3 relatives (ages 48-50), and n=10 clinicians (ages 37-60)  Other demographics:  Youth/adults: 62% female  Relatives: 100% female  Clinicians: 90% female | ***Acceptability Outcomes/Other Considerations:***  -mHealth app found to be useful in recognizing precipitating stressors leading to a crisis and identifying coping strategies to mitigate crisis  -Wide range of differences found in user engagement and reliance of supportive networks, with some concerns about sharing information with relatives and clinicians especially in non-acute states  -Involvement of clinicians to develop safety plan found to increase motivation to use the app; clinician support may provide an opportunity to learn/review strategies in non-acute states as distressed states may pose challenges in self-awareness, problem-solving, and communication  -Helpful app features included: prewritten messages, minimal clicks, speed dialing, electronic “Hope Box”  ***Future Directions:***  -Explore the utility of user app set-up guidelines via web-based educational tools/resources |
| Jeong et al., 2020, *Brake of My Mind* [57] | South Korea | **Research Designs:** (1) Usability and (2) Effectiveness (pilot one-group pretest-posttest)  Developed a mHealth safety plan app (Brake of My Mind) for adolescent suicide attempt survivors based on the Theory of Planned Behavior framework (Study 1); evaluated the feasibility of the app with target users in small pilot study (Study 2) | **Sample Study:** (1) Heuristic evaluation with n=5 experts in nursing informatics, computer science, and digital media design (ages 41-49)  Other demographics: 80% male, 20% female  (2) User evaluation with n=3 youth (ages 12-16) with history of suicide attempt and in case management services and n=6 healthcare professionals (ages 29-41) with experiencing working with adolescent suicide attempt survivors  Other demographics: Youth: 75% female, 25% male  Clinicians:83% female, 17% male | ***Usability/Effectiveness (TPB Outcomes):***  -Heuristic evaluation: No/minimal usability problems; app revisions made to user interface, added help feature  -User evaluation: task difficulty varied among youth and adult users, with responses ranging from easy or very easy (n=4), average (n=5), and difficult (n=1); time completion for each task varied, with youth taking longer to complete (X=29.50 minutes all timepoints vs. 20 minutes for adults); overall usability score was 70, which exceeds “average usability” for technological applications; youth group had a higher usability score than professionals suggesting high efficiency, effectiveness, and satisfaction  -TPB constructs: suicide intention decreased immediately following app usage and at follow-up (7-days); attitudes toward suicide attempts decreased following app use and remained the same at follow-up (lower than baseline); perceived behavioral control following app usage remained the same as baseline, but decreased at follow-up suggesting that making an attempt may be more difficult to carry out; no changes found in subjective norms  ***Future Directions:***  -Improved research design with larger sample to examine clinical outcomes  *-*Future evaluations should include a longitudinal approach that analyzes potential impacts of various participant characteristics |
| Kennard et al., 2015, *Unnamed* [43] | U.S. | **Research Design:** (1) Formative Interviews  Conducted semi-structured interviews to guide the development of mHealth safety planning app for youth hospitalized for suicidality (explored care transitions, treatment targets, and safety planning); assessed initial acceptability of a mHealth app for safety planning | **Study Sample:** (1) Interviews with n=10 youth (ages 14-17, X=15.5 [.92]) at risk of suicide, n=10 parents, and n=9 mental health clinicians experienced in the management of suicidal youth  Other demographics: Youth: 80% female; 90% White  Parents: 90% female and White  Clinicians: 67% female | ***Formative/Acceptability Outcomes:***  -Factors influencing successful care transitions included open communication, timely/rapid transitions, creation of transition plans and safety plans, and social support. Primary barriers included limited time, lack of availability of clinicians, poor relationships with providers, and low motivation  -Barriers influencing the use of safety plans included lack of motivation, unhelpful skills/content obtained in safety plan, accessibility, not wanting others to be involved, and emotional dysregulation  -Both youth and parents noted the importance of parental involvement in developing a safety plan; key treatment targets to focus on included sleep and sobriety skills and social support strategies; personalization features could target specific skills  -Majority of participants responded favorably to using a safety planning app; parents reported the app would be a convenient/accessible way to ensure safety; youth felt they would be comfortable using the app and that it would be helpful  -Drawbacks of using a mHealth app included privacy/confidentiality, phone restrictions, and limited customization  ***Future Directions:***  -Future development of the mHealth app to incorporate strategies to manage distress, integrate tools to improve provider-patient (family) communication, target symptoms such sleep, drug/alcohol use, and social support, and incorporate motivation interviewing strategies to enhance motivation to use the app  -Pilot and RCT to examine use, access, and effectiveness of the mHealth app as youth transition from inpatient to outpatient services |
| Larkin et al., 2023, *ED-SAFE* [66] | U.S. | **Research Designs:** (1) Formative and (2) Usability Interviews  Described iterative development process for creating ReachCare, an emergency department (ED) safety planning app and a patient mHealth app version of ED-SAFE (i.e., a paper-based safety plan + behavioral health referral resources); usability testing with two sample of patients in an ED setting (findings from study 1 used to refine the mHealth app) | Study Sample: (1) App design/iterative feedback with n=7 subject matter experts (n=3 clinicians and n=4 suicidologists) and 6 individuals with lived experience of suicidality (age range= 18-73). Usability evaluation with n=2 emergency department outpatients.  Other demographics (individuals with lived experiences): 50% male; 83% White  (2) Usability testing with patients at risk of suicide: Cycle 1: n=9, mean age 29.2 (SD=12.9); Cycle 2: n=5, mean age 26.4 (SD=8.4) discharged from ED  Other demographics: Cycle 1: 67% men, 33% women; 78% White, 11% Black, 11% 1+ race; 56% Hispanic  Cycle 2: 40% women, 40% non-binary, 20% men; 80% White, 20% 1+ race, 40% Hispanic | ***Formative/Usability Outcomes:***  -Initial app design ideas centered on creating a mHealth safety planning app aimed to reduce cognitive effort and facilitate a calm, caring, supportive, and welcoming space for the end-user (design objectives formed from interviews with subject matter experts)  -Lived experience feedback validated initial app design objectives (e.g., clear and caring app design, use of chatbots, introductory videos); recommend using a tile-based user interface  -Cycle 1 usability testing: usability scores acceptable; positive feedback regarding interface and functionality. Recommended changes to ‘distractions’ page and help tab  -Cycle 2 usability testing: usability scores acceptable. Recommended adding ability to share plan with clinicians and adding additional ‘distractions’ (i.e., videos)  -40% downloaded app post-discharge, participants (n=2) had positive experiences with app; app usage ranged from one time to a few times  ***Future Directions*:**  -Future studies to include more personalized features to address unique needs of different populations; improve user engagement  -mHealth app design decisions should incorporate qualitative interviews with standardized usability and engagement measures |
| Meier et al., 2022, *SERO* [58] | Switzerland | **Research Design:** (1) Focus Groups (Formative)  Described development of the SERO-app, a mHealth app that integrates safety planning and tracking of mood and suicidal thoughts via a suicide risk assessment (an adapted version of the PRISM-S) and self-reflection questionnaire; formative usability test | **Study Sample:** (1) Formative focus groups with 11 psychiatric inpatients  Demographics: none reported | ***Formative/Other Considerations:***  -Described the conceptual architecture, app modules, and interface design for two versions of the SERO-app: (1) Version 1, to be used with suicidal persons only and (2) Version 2, to be used by family members or loved ones (data sharing)  -Patients felt supported by the mHealth app. Desired emergency button in app design  -Patients can visually assess and monitor risk for suicide via app  -Data stored from self-assessments could be share in future therapy sessions  ***Future Directions*:**  -Future studies to assess utility/effectiveness of SERO V1.0  -App development: push-notifications of subsections of safety plan |
| Melia et al., 2023, *SafePlan* [59] | Ireland | **Research Designs:** (1) Pilot RCT Research Protocol Only  Developed mHealth app (SafePlan) study protocol to: (1) evaluate the feasibility and acceptability of using the mHealth app as an adjunct to treatment as usual in a mental health setting, (2) assess feasibility of study protocols and determine sample size for future RCT, (3) compare clinical outcomes between mHealth app condition + treatment as usual vs. paper safety plan + treatment as usual, and (4) conduct an economic analysis of the mHealth intervention | **Targeted Sample (future):** (1) youth and adult clients (aged 16-35) with current suicide ideation and/or history of nonsuicidal self-injurious behavior or suicide attempt receiving secondary-level mental health services (n=40 each arm); (2) clinicians providing mental health services to youth/adult clients at risk of suicide (n=12) | ***Key Findings:*** Not applicable. Future work.  ***Considerations:***  -In addition to using standardized measures, researchers plan to conduct qualitative interviews to further assess acceptability of study procedures and better understand experiences/barriers related to participation in both conditions |
| Melvin et al., 2019, *BeyondNow* [60] | Australia | **Research Design:** (1) Feasibility and (2) Effectiveness (two month open-trial, one group pre-posttest)  Examined the feasibility of integrating the mHealth app, BeyondNow into mental health services; examined the effectiveness of app with TAU (App+TAU) in reducing suicide ideation and increasing suicide-related coping and resilience | **Study Sample:** (1) n=36 youth and adult clients (ages 16-42; M=19.89 [6.04]) receiving treatment for suicide risk completed measures at baseline and posttest (8-weeks following intervention). Follow-up n=22  Other demographics:  66.7% female | ***Feasibility/Effectiveness Outcomes and Considerations:***  -Most participants accessed the app during the study either to edit their plan (77%) or to view it to help manage their suicide ideation (82%) or a suicidal crisis (68%); majority (>90%) found the app easy to navigate  -All participants at follow-up reported they would recommend the app to a friend  -Intervention was associated with users increased knowledge and confidence in implementing coping strategies to help with suicide ideation  -Demonstrated a significant decrease in both severity and intensity of suicide ideation and an increase in suicide-related coping across study trial; no statistically significant changes found in suicide resilience  -High attrition, which could have impacted findings through non-response bias  ***Future Directions:***  *-*App may reduce suicide ideation via coping, need to examine this relationship in RCT  -Examine relationship between individual app usage and effect on suicide-related coping and frequency/intensity of suicidal ideation (dose effect) |
| Muscara et al., 2020, *BeyondNow* [61] | Australia | **Research Design:** (1) Feasibility and (2) Open-trial Study Design  Examined the feasibility (uptake, retention, adherence) and acceptability of using two different mHealth apps in conjunction (i.e., BeyondNow [digital safety plan] and BlueIce [personalized toolbox of strategies focused on mindfulness and relaxation activities and automatic safety checks]) over the course of six weeks; examined the effectiveness of apps in reducing self-harming thoughts and behaviors and increasing suicide resilience. A follow-up study to [62]. | **Study Sample:** (1) n=17 youth clients (ages 13-18; M=15.50 [1.28]) at risk of suicide discharge from a pediatric inpatient mental health unit  Other demographics:  80% female | ***Feasibility/Other Outcomes and Considerations:***  -Feasibility: 85% retention rate; recruitment rate of 41.4% of eligible clients (above CONSORT guidelines); among youth experiencing a suicidal crisis post-discharge, 73.5% used at least one of the apps during a suicidal crisis (adherence below CONSORT guidelines)  -Acceptability: Participants used both apps at least once post-discharge (58.8% BeyondNow and 35.3% BlueIce), with majority using at least once during a crisis; over 50% felt comfortable using both apps and were satisfied with functionality; 35% would use both apps in the future  -Majority of participants believed the apps were not helpful managing symptoms during a crisis (52.9% BeyondNow; 41.2% BlueIce) and did not believe/unsure if apps could keep them from harming oneself (47.1% both)  -No significant decreases found for self-harm thoughts and behaviors; a significant increase found in emotional stability factor (one sub-scale within suicide resilience constructs)  ***Future Directions:***  *-*Clinical trial needed to examine impact on self-harming behaviors and suicide resilience  -Further studies should examine different clinical and technological approaches and influence on treatment engagement and clinical outcomes; examine health economic benefits for using mHealth apps, including impact on hospital admissions |
| Nuij et al., 2018, *BackUp* [42] | Netherlands | **Research Design:** Research Protocol Only for Single Cohort Design  Developed study protocol to assess feasibility (useability, uptake, satisfaction) of a mHealth app (BackUp) and daily self-monitoring (ecological momentary assessment [EMA]) app in routine clinical practice; validate psychological processes posited by the Integrated Motivational-Volitional (IMV) Model and identify pathways to suicidal behavior and profiles of suicidal individuals | **Targeted Sample (future):** (1) adults (over 18) with major depression or dysthymia disorder, with suicide ideation (n=80) receiving outpatient or day-care from mental health organizations | ***Considerations:***  -Self-monitoring of symptoms in real-time can be used to direct clinical treatment  -Combination of both safety planning with EMA may decrease suicidal symptoms  -Understanding psychological processes and stages of suicide pathways (and profiles of subgroups of individuals at risk of suicide) can guide suicide prevention and clinical interventions  ***Future Directions*:**  -Integrate EMA within safety planning app (if found to be feasible); combination of both features could lead to ecological momentary intervention (EMI) where real-time symptoms could prompt safety plan |
| Nuij et al., 2022, *BackUp* [62] | Netherlands | **Research Design:** Three-month Feasibility  Assessment (usability, uptake, satisfaction) of a mHealth app (BackUp) and daily self-monitoring (ecological momentary assessment [EMA]) app as treatment components in routine clinical practice. A follow-up study to [42]. | **Study Sample:** (1) adult patients (n=17) with major depression or dysthymia disorder, with suicide ideation receiving outpatient services from three mental health facilities (ages 20-50; M=32.12 [9.16]). Follow-up n=12  Other demographics:  52.9% male  47.1% female  (2) Clinicians (n=7) providing clinical services to adults diagnosed with major depression or dysthymia disorder, with suicide ideation  Other demographics:  57.1% female  42.9% male | ***Feasibility Outcomes and Considerations:***  -Most participants completed mHealth safety plan with clinicians, met minimum uptake rate (>75%)  -At follow-up timepoints (4 and 12-weeks post-baseline), patient usability and satisfaction scores exceeded acceptability standards  -Majority of patients found the BackUp app easy to use, convenient, and useful. 29% did not use the app during the study period  -Clinicians reported difficulties using a phone to collaborate with patients to complete safety plans; integration of the mHealth app in routine practice did not meet minimum uptake standards (33.3% vs. 75%). Clinicians noted the need for mHealth apps to be part of the technological infrastructure of the institution  -Interactive options and quick access were noted as benefits among clinicians  ***Future Directions:***  -Implementation studies are needed to explore how mHealth apps can be embedded in the technological infrastructure of mental health/healthcare settings |
| O'Grady et al., 2020, *SafePlan* [63] | Ireland | **Research Designs:** (1) Formative and (2) Usability Survey and Focus Groups  Described development of SafePlan, a mHealth app that integrates safety planning and DBT approaches (involved stakeholder feedback in design); usability and functionality testing with sample of youth (not at-risk of suicide). Goal not to evaluate content of app but usability and functionality | **Study Sample:** (1) App design/iterative feedback with 15 health care professionals who provided mental health treatment to youth or adult clients.  Other demographics: none reported.  (2) Usability and functionality testing with community sample of 18 students (ages 14-16) with technology experience.  Other demographics: none reported. | ***Formative/Usability Outcomes:***  -In addition to Safety Plan and DBT Diary features, other app features created included a chart button where safety plans or diary cards could be printed or saved as PDF or CSV file (to be shared with clinicians during session), tracking button to monitor use of diary items and changes made to safety plan, and notification system to update behaviors and share data (in person sharing)  -Safety Plans and Diary Cards not shared or generated via external server or cloud portal which addressed privacy and security-related concerns  -Overall usability score was 71.85, exceeds “marginally acceptable” score for usability of technological applications; highest scores reflected ease of use, confidence using app, coherent integration of features (sections) in app  -Other impressions of app (qualitative): simplicity of design, visual appearance, and privacy safeguards for storage of data and access (use of password)  -Recommendations for app refinement: tutorial/directions on how to use the app; motivational messages; addition of calming sounds or music; reduce amount of content/information displayed (may overwhelm end users)  ***Future Directions*:**  -Future studies to include target population (i.e., clients at risk of suicide), particularly RCT to examine feasibility and acceptability of app (as an adjunct to therapy) and data collection methods (EMA; sharing of information with clinicians)  -App development: in-app warning regarding usage (to be used after orientation session) and automated notifications for users to seek professional help |
| Pauwels et al., 2017, *BackUp* [65] | Belgium | Research Design: (1) Acceptability and (2) Effectiveness (one group pre-posttest)  Described development of BackUp, mHealth app designed to support users at risk of suicide and their support network; examined usability/acceptability with expert panel and end users; suicide ideation scores measured before and after testing period (1-week) with end-users | Study Sample: (1) Evaluation measure completed by expert panel of suicide prevention experts and mental health professionals (n=8) and 21 adults (ages 18-54) with suicide ideation (2)  same 21 adults completed pre-post measure for suicide ideation (Beck Scale for Suicide Ideation)  Other demographics: Includes male and female end-users, but exact count is unknown | *Acceptability Outcomes/Other Outcomes and Considerations:*  -Acceptability (expert panel): Majority of participants (over 85%) had positive perceptions in terms of look/feel and initial impressions; 4 tools within app were perceived to be mostly useful; “Back-up Cards” (coping cards) deemed to be least helpful by majority of participants; all participants felt the app would be helpful to people experiencing suicidal thoughts  -Acceptability (end users): 95% of participants used the app at least one time during the testing period, individuals with high suicide ideation scores used the app several times or daily; 41% reported the app helped with suicidal thoughts (41% had mixed feelings; 18% did not agree). Most helpful features of the app included safety plan (45.5%) and “Back-up Box” (support network; 18.2%). Least helpful features: Back-up Cards (coping cards; 27.3%), My BackUps (support network; 22.7%) and Backup Box (reasons for living; 22.7%). 63.6% would use the app daily (18.2% had no opinion/would not use, respectively).  -Reduction in suicide ideation scores from baseline, but not statistically significance  *Future Directions:*  *-*Need to examine effectiveness of suicide prevention apps, especially to reduce suicide behaviors |
| Skovgaard Larsen et al., 2016, *MYPLAN* [64] | Denmark | **Research Design:** (1) Acceptability  Described MYPLAN, an mHealth app designed to support users at risk of suicide by facilitating the use of an electronic safety plan; provided clinician and user perceptions/experiences using the app | **Study Sample:** (1) case reports from adult clients at risk of suicide (age range 23-52) and clinicians collaborating with suicidal clients at suicide prevention clinics (unknown age)  Other demographics: Included male and female clients, unknown sample size; other demographics not reported | ***Acceptability Outcomes/Other Considerations*:**  -Provided description of mHealth app components; app was designed to be a supplement to treatment and for individuals who prefer self-help methods or who cannot access therapeutic supports  -App can be translated to other languages for diverse populations  -Initial results from case reports suggest the app was well received by clients at risk of suicide and clinicians; easy to use and app being used in crisis situations (note: limited study details of case report methodology)  -Involvement of clinicians to develop safety plan may increase intentions to use the app, especially for individuals who have limited familiarity with apps (e.g., older adults)  ***Future Directions:***  -Future iterations of the app include tracking and sharing capabilities  -Future RCT to study effectiveness of the intervention, evaluate changes in suicidal behavior |
