# Supplementary material for: Translating Suicide Safety Planning Components Into the Design of mHealth App Features: Systematic Review
Source: JMIR Ment Health. 2024 Mar 28;11:e52763. doi: 10.2196/52763 (PMC11009854; doi:10.2196/52763)
Supplement: Multimedia Appendix 4 [file mental_v11i1e52763_app4.docx]

**Appendix 3.** Critical Appraisal Results

Quasi-Experimental Study Appraisal Checklist (assessed using Tufanaru et al., 2020)

| **Author/Year** | **Q1** | **Q2** | **Q3** | **Q4** | **Q5** | **Q6** | **Q7** | **Q8** | **Q9** | **Overall** |
| --- | --- | --- | --- | --- | --- | --- | --- | --- | --- | --- |
| Jeong et al., 2020 [57] | Y | Y | N/A | N | Y | N/A | Y | Y | Y | Include |
| Melvin et al., 2019 [60] | Y | Y | N/A | N | N | N/A | Y | Y | Y | Include |
| Muscara et al., 2020 [61] | Y | Y | N/A | N | N | N/A | Y | Y | Y | Include |
| Nuij et al., 2022 [62] | Y | Y | N/A | N | Y | N/A | Y | Y | Y | Include |
| Pauwels et al., 2017 [65] | Y | Y | N/A | N | N | N/A | Y | Y | U | Include |

Y: Yes; N: No; N/A: Not applicable; U: Unclear

Qualitative Study Appraisal Checklist (assessed using Lockwood et al., 2015)

| **Author/Year** | **Q1** | **Q2** | **Q3** | **Q4** | **Q5** | **Q6** | **Q7** | **Q8** | **Q9** | **Q10** | **Overall** |
| --- | --- | --- | --- | --- | --- | --- | --- | --- | --- | --- | --- |
| Buus et al., 2020 [56] | N | Y | Y | Y | Y | N | U | Y | Y | Y | Include |
| Kennard et al., 2015 [43] | N | Y | Y | Y | Y | N | U | Y | Y | Y | Include |
| Skovgaard Larsen et al., 2016 [64] | N | U | U | U | U | N | U | U | U | U | Include |
| Meier et al., 2022 [58] | N | U | U | U | U | U | U | U | U | U | Include |

Y: Yes; N: No; N/A: Not applicable; U: Unclear

Mixed Method Studies: Combined Quasi-Experimental and Qualitative Study Appraisal Checklists (assessed using Lockwood et al., 2015 and Tufanaru et al., 2020)

| **Author/Year** | **Q1** | **Q2** | **Q3** | **Q4** | **Q5** | **Q6** | **Q7** | **Q8** | **Q9** | **Q10** | **Overall** |
| --- | --- | --- | --- | --- | --- | --- | --- | --- | --- | --- | --- |
| Larkin et al., 2023 [66] QNT | Y | Y | N/A | N | N | N/A | Y | Y | Y | - | Include |
| Larkin et al., 2023 QUL | Y | Y | Y | Y | Y | Y | Y | Y | Y | Y |  |
| O’Grady et al., 2020 [63] QNT | Y | Y | N/A | N | N | N/A | Y | Y | Y | - | Include |
| O’Grady et al., 2020 QUL | Y | Y | Y | Y | Y | Y | Y | Y | Y | Y |  |

Y: Yes; N: No; N/A: Not applicable; U: Unclear; QNT: Quantitative Checklist; QUL: Quantitative Checklist
